# Antitumor and antimetastatic effects of dietary sulforaphane in a triple-negative breast cancer models

**DOI:** 10.1038/s41598-024-65455-w

**Published:** 2024-07-11

**Authors:** A. Pogorzelska, M. Świtalska, J. Wietrzyk, M. Mazur, M. Milczarek, K. Medyńska, K. Wiktorska

**Affiliations:** 1 Department of Biomedical Research, National Medicines Institute, Chełmska 30/34, 00-725 Warsaw, Poland; 2 Laboratory of Experimental Anticancer Therapy, Hirszfeld Institute of Immunology and Experimental Therapy, Polish Academy of Sciences, Rudolfa Weigla 12, 53-114 Wrocław, Poland; 3 Department of Chemistry, University of Warsaw, Ludwika Pasteura 1, 02-093 Warsaw, Poland; 4 Department of Physics and Biophysics/Institute of Biology, Warsaw University of Life Sciences, Nowoursynowska 159, 02-776 Warsaw, Poland

**Keywords:** Sulforaphane, Triple-negative breast cancer, Dietary doses, Metastasis, Breast cancer, Drug discovery

## Abstract

Triple-negative breast cancer (TNBC) represents aggressive phenotype with limited treatment options due to the lack of drug targets. Natural compounds are extensively studied regarding their potential to alter the efficacy of cancer treatment Among them sulforaphane – an isothiocyanate of natural origin, was shown to be a hormetic compound, that may exert divergent effects: cytoprotective or cytotoxic depending on its concentrations. Thus, the aim of this study was to determine the effect of its low, dietary concentrations on the proliferation and migration of the TNBC cells in the in vivo and in vitro 2D and 3D model. Results of the in vivo experiment showed up to 31% tumor growth inhibition after sulforaphane treatment associated with lowered proliferating potential of cancer cells, reduced areas of necrosis, and changed immune cell type infiltration, showing less malignant type of tumor in contrast to the non-treated group. Also, the study revealed that sulforaphane decreased the number of lung metastases. The in vitro study confirmed that SFN inhibited cell migration, but only in cells derived from 3D spheroids, not from 2D in vitro cultures. The results show a specific role of sulforaphane in the case of cells released from the TNBC primary tumor and its environment.

## Introduction

Many phytochemicals exhibit a non-monotonic dose/concentration response, termed biphasic dose–response, and are classified as hormetic compounds, i.e., they produce biologically opposing effects at different doses^1,2^. Most commonly, they have a stimulatory effect at low concentrations, whereas they show an inhibitory effect at high concentrations^3^. One of such compounds is a phytochemical sulforaphane (1-isothiocyanato-4-(methylsulfinyl)-butane, SFN).

SFN is an isothiocyanate, whose precursor glucoraphanin is found in the vegetables from the Brassicaceae family, such as broccoli and cabbage. Glucoraphanin is hydrolyzed by an enzyme myrosinase to an active compound SFN^4^.

SFN at high concentrations is known for its anti-tumor features. At concentrations of 10–50 µM, it acts as a pro-apoptotic and pro-autophagy agent in various malignancies^5^. It has been shown that SFN can promote cell cycle arrest^6^. SFN also emerges as a tumor microenvironment modulator^7^. It has the potential to influence cancer stem cells (CSCs), a cancer cell population, therapy-resistant and responsible for cancer relapse^8,9^. More importantly, SFN is tested as a co-adjuvant in chemotherapy, being proposed a novel combination treatments, where SFN strengthens the action of the drug while minimizing side effects on normal cells. Hence, SFN is a promising co-adjuvant agent in preventing drug resistance, metastasis and tumor relapse^10^.

Numerous studies have shown that SFN at higher concentrations is an anticancer agent in various cancer types, mainly in breast cancer^11^. Breast cancer stands as a predominant cause of mortality among women on a global scale. Triple-negative breast cancer (TNBC) is a subtype of breast cancer characterized by lack of hormone receptors, and human epidermal growth factor receptor 2 expression, a heterogeneous and aggressive phenotype with limited treatment options. Current therapeutic approaches remain inadequately effective. A promising approach to cancer prevention focuses on a healthy lifestyle that includes an anti-inflammatory diet and stress reduction. Thus, natural compounds, and in particular SFN, are of interest to prevent disease and possibly assist treatment, when chemotherapeutics are still the best possible treatment option^3,12^. These compounds are recognized for their dual attributes as chemopreventive agents, capable of impeding cancer onset, and as anticancer agents for treating established malignancies.

SFN at low concentrations is also a well-known chemo-preventive agent in breast cancer (0.5–10 µM). The mechanism of SFN action is multidirectional and includes increased levels of detoxification enzymes, decreased enzymatic activity of P450 cytochrome, reduced cancer cell proliferation through inhibition of the cell cycle, induction of apoptosis and autophagy, as well as elimination of cancer stem cells^13^. SFN is also one of the most well-known Nrf2-ARE pathway agonists, an up-regulator of phase II detoxification enzymes, and an anti-inflammation agent^14^.

On the other hand, SFN showed biphasic effects on cancer cell growth, cancer cell migration, and angiogenesis. Effect of SFN at lower doses could be different in normal and tumor cells^1^. At low concentrations, SFN promotes pro-survival autophagy or remains latent, which taking into account its low bioavailability in human plasma after consumption could have serious side effects on developing tumor or therapy effectiveness^15,16^. It was hypothesized that, while in a healthy organism, this effect of SFN is beneficial, in a developing cancerous tumor action of SFN, especially at low concentrations, can induce the opposite effect: it can promote tumor formation^9,17^. It was already shown, that the use of antioxidant supplements during chemotherapy, as well as iron and vitamin B12, may increase the risk of breast cancer recurrence and mortality^18^ . In the case of SFN, its serum concentrations after broccoli consumption were reported to reach 0.94–2.27 μM^19^. Thus, testing a low dosage of SFN is of great importance, particularly when dietary supplements are commonly administered without advisory^3^.

In this article, the focus was on the role of low SFN concentrations, corresponding to dietary levels in breast cancer. The aim was to test the such SFN concentration effect on tumor development: proliferation and migration of the TNBC cells in the in vivo and in vitro model.

## Material and methods

### In vivo antitumor efficacy studies

All animal procedures were carried out in accordance with the Directive of The European Parliament on the protection of animals used for scientific purposes (2010/63/EU of European Parliament) and with the consent and the experimental protocols approval of the Local Ethics Committee for Experiments with the Use of Laboratory Animals (permit no. 34/2019 received 19 June 2019) based at the Hirszfeld Institute of Immunology and Experimental Therapy in Wrocław,12 Weigla St., 53-114 Wrocław. The study was reported in accordance with ARRIVE guidelines.

A 4T1 implanted murine breast tumor model was established in female BALB/c mice (Medical University of Białystok, Poland). The 2 × 10^5^ 4T1 cells from in vitro culture in 0.05 mL PBS were injected into the mammary fat pad. Ten days after injection, when the tumor mean volume was 100 mm^3^, mice were divided into two groups of eight mice. Each group was administered intraperitoneally (IP) with injection water (control group, 0.1 mL/10 g) and SFN 0.026 mg/kg on days 10, 16 and 23 (the SFN dose corresponds to dietary concentrations observed after broccoli consumption). This route of administration was chosen because of relatively hydrophobic nature of SFN and higher absorption rate comparing to intravenous administration. To monitor the cytotoxicity of the administered dose, tumor sizes—width (a) and length (b)—were measured using calipers (CD-15DCX type, Mitutoyo Corp., Japan), and body weight was measured using an analytical balance (Mettler Toledo, Poland) three times per week. Antitumor activity was evaluated in terms of tumor volume (TV) and calculated according to the following formula:

$$TV\left[{mm}^{3}\right]=\left({a}^{2}\times b\right)/2,$$

where a and b are the axes of tumors.

Mice were sacrificed on day 28. During the autopsy, organs such as heart, liver, lungs, and spleen had been weighed. Primary tumors, lungs, and blood were collected for further analysis.

### Histopathology and immunohistochemistry analysis

The primary tumors were examined histologically. Tissues were fixed in 10% buffered formalin, embedded in paraffin, sectioned, and stained. To evaluate the mitotic activity, the sections were stained with hematoxylin and eosin. In the areas of viable tissue, mitotic indices were counted, i.e. the average number of mitoses in ten fields of view at a lens magnification of 20x. To evaluate the proliferating index, immunohistochemical staining was performed. Tissue sections were stained using anti-Ki-67 Rabbit Monoclonal Primary Antibody (Roche) on an automated immunohistochemical stainer. Histopathological examination was performed with a 40× lens magnification in ten fields of view. The result was given as % positive cells in the pool of 1000 cells evaluated. Observations were performed on an Axiolab A5 microscope (Zeiss, Germany).

### The antimetastatic activity of tested formulations

Lungs were collected in formalin to count metastatic foci. The number of 4T1 metastases in the lungs in the control group and after the administration of SFN was counted.

### Blood analysis

Blood was collected from mice to vials with EDTA and blood morphological analysis was performed using a hematological analyzer (Mythic18, Cormay Diagnostics, Poland), then the blood was centrifuged (15 min, 2500×*g*, 4 °C) for plasma isolation and stored at −80 °C. Biochemical analysis was performed in the plasma, and the following biochemical parameters were determined: alanine aminotransferase (ALT), aspartate aminotransferase (AST), creatinine, and creatinine kinase (CK) were analyzed using a biochemical analyzer (Cobas c111, Roche, Swiss).

### Cell culture

MDA-MB-231 human breast adenocarcinoma and 4T1 mouse mammary carcinoma, which are the model lines for TNBC research, were purchased from the American Type Culture Collection (ATCC, USA). Cell culture was monitored regarding mycoplasma contamination using a luminescence assay MycoAlert^®^ Mycoplasma Detection Kit (Lonza, Switzerland). MDA-MB-231 cells were cultured in IMDM (Biowest) supplemented with fetal bovine serum (10% v/v), 100 U/mL penicillin, 100 µg/mL streptomycin, 0.250 µg/mL amphotericin B (Biowest) and non-essential amino acids (0.1 mM) (Sigma Aldrich). 4T1 cells were cultured in RPMI-1640 (Gibco) supplemented with 10% FBS (GE Healthcare), 1 mM Sodium Pyruvate, 3.5 g/L d-( +)-glucose, 100 µg/mL streptomycin (Sigma-Aldrich), 100 U/mL penicillin (Polfa Tarchomin S.A.). Cells were cultured in sterile flasks at 37 °C in a humidified incubator with a 5% carbon dioxide atmosphere. After reaching 80% confluence, cells were trypsinized with trypsin/EDTA solution (Sigma Aldrich) and diluted in fresh medium to keep the continuous cultivation.

For the 3D culture, MDA-MB-231 cells were seeded in 96-well U-shaped Ultra Low Attachment plates (Corning, USA) at a density of 8000 cells/well. The plate was centrifuged for 5 min 200*g* and incubated at 37 °C, 5% CO_2_. After 24 h medium containing collagen was added to obtain a 3,5 µg/ml final concentration of collagen and centrifuged for 3 min 100*g*. Cells were cultured for another 6 days to form spheroids.

### MTT cytotoxicity assay

The viability of cells seeded 2D was determined using a colorimetric MTT assay. Cells were seeded in 96-well plates (Cytogen, Zgierz, Poland) at a density of 40,000 cells/mL (MDA-MB-231) After 24 h, a medium containing the increasing concentrations of SFN (0.06–250 µM) was added to the cells, and plates were incubated for 72 h. After that, 50 µL/well of MTT solution (0.25 mg/mL PBS) was added to each well. After 3 h of incubation at 37 °C, medium was aspirated and 200 µL of 2-propanol was added to dissolve the formazan crystals. Absorbance was measured at 570 nm for MTT determination on a BioTek PowerWave XS microplate reader (Agilent, Santa Clara US).

### PrestoBlue cytotoxicity assay

The viability of cells seeded 3D was determined using a colorimetric PrestoBlue^®^ assay. After spheroid formation, as previously described in a 2.5 section, a medium containing increasing concentrations of SFN (0.5–250 µM) was added to the cells, which were further incubated for 72 h. At the end of the experiment, Presto Blue stain was added in a 1:200 ratio and incubated for 1 h at 37 °C. After incubation, the fluorescence was measured at 560/590 nm using an Infinite M100 Pro microplate reader (Tecan, USA).

### Live/dead viability assay

Cells were stained using Fluorescein diacetate (FDA) and Propidium Iodide (PI) to visualize live and dead cells in the spheroids after treatment with SFN. After spheroid formation—as previously described in a 2.5 section—a medium containing increasing concentrations of SFN (0.5–250 µM) was added to the cells, which were further incubated for 72 h. At the end of the treatment, cells were stained with 0.05 µg/ml FDA and 7 µg/ml PI and incubated for 30 min at 37 °C. The results were visualized using confocal microscopy Fluoview 500/IX70 (Olympus, Tokyo, Japan).

### Wound healing assay

A wound healing assay was performed to evaluate cell migration potential change after treatment. MDA-MB-231 cells from 2D culture were seeded into the 8-well plates (Labtek, Thermofisher) at the density 6 × 10^4^ cells cells/ml. When the cells were approximately 80% confluent, the scratch was made, and cells were treated with different concentrations of SFN. Similarly, the assay was performed on the MDA-MB-231 cells after 10 days of 3D culture (Huang et al., 2020). The spheroids were removed from the 96-plate, digested with trypsin/EDTA, and seeded (6 × 10^4^ cells/well) into the eight-well plates (Labtek, Thermofisher) under the same 2D culture conditions until the cells reached 80% confluence. The scratch was made, and cells were treated with different concentrations of SFN (0.5; 1; 10 µM). The size of the scratch was visualized using a confocal microscope Olympus Fluoview 500/IX70 after 0 h, 4 h, 24 h and 48 h of treatment. The results were analyzed using ImageJ software. The wound closure was calculated according to the formula:

$$\text{\% wound closure}={({\text{A}}_{0} - {\text{A}}_{\text{t}})/\text{A}}_{0}\times 100\text{\%},$$

where A_0_ is the area of the wound measured immediately after scratching (time zero), and A_t_ is the area of the wound measured h hours after the scratch is performed.

### Statistical analysis

The statistical analysis was performed in GraphPad Prism Software (Graph-Pad Software, San Diego, US) using an appropriate test per experiment. p < 0.01 was considered statistically significant.

### Ethics approval

All animal procedures were carried out in accordance with the Directive of The European Parliament on the protection of animals used for scientific purposes (2010/63/EU of European Parliament) and with the consent of the Local Ethics Committee for Experiments with the Use of Laboratory Animals based at the Hirszfeld Institute of Immunology and Experimental Therapy in Wrocław, 12 Weigla St., 53-114 Wrocław. Number of permit for animal experiments 34/2019 received 19 June 2019. The study was reported in accordance with ARRIVE guidelines.

## Results

### Antitumor effectiveness in vivo of small doses of SFN

**Figure 1:**
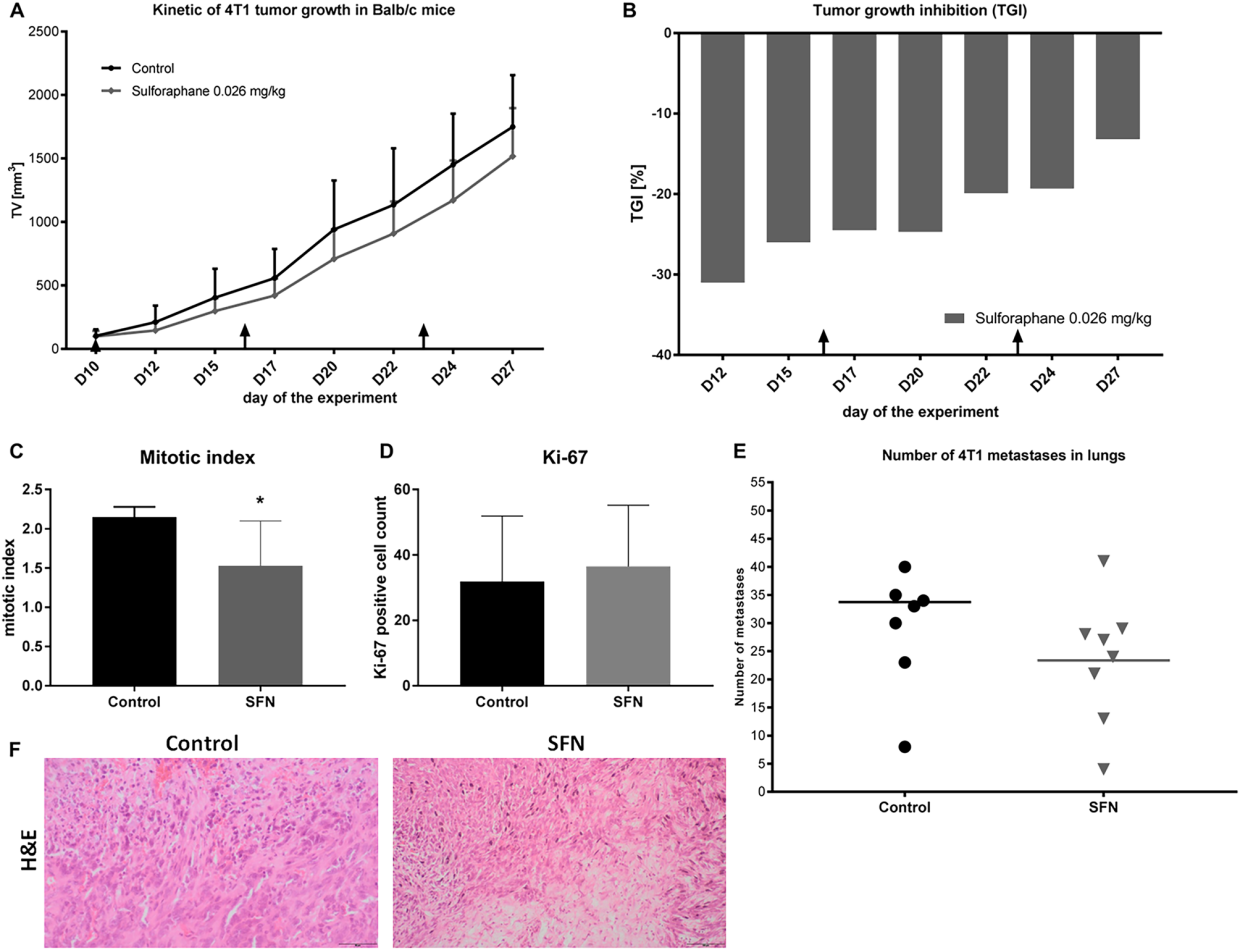
The effect of 0.026 mg/kg SFN on primary tumor growth, histology, and metastases. (**A**) Tumor growth curves—kinetic of 4T1 tumor growth in BALB/c mice—tumor volume (TV) (mm^3^), (**B**) tumor growth inhibition (TGI) after SFN treatment calculated in comparison to the control group. The arrows indicate the administration of SFN or injection water at days 10, 16, and 23, (**C**) mitotic index and (**D**) Ki-67 positive cell count calculated from the histological analysis of mouse tumor sections, (**E**) number of metastases in lungs, the line corresponds to mean number of metastases in lungs. All data are presented from the control group and after administration of SFN (0.026 mg/kg) on days 10, 16 and 23 of the experiment as mean ± SD (n = 8). *p < 0.05 vs control. (**F**) Hematoxylin & eosin histochemical staining of tumor sections. Scale bar 100 µm.

An in vivo experiment on a mouse model of TNBC was performed. The mice received intraperitoneally low concentrations of SFN (0.026 mg/kg) on days 10, 16, and 23 of the experiment. The tumor growth kinetics were observed for 27 days. On the first days of the experiment, SFN inhibited 4T1 tumor growth at about 25–31%. Later, the effect decreased to about 13–20% tumor growth inhibition (TGI) (Fig. 1A, B).

The histological evaluation of tumors was carried out to assess the changes in tumor-forming cells after SFN administration, leading to inhibition of tumor growth. Mitotic Index (MI) and Ki-67 positive cells were counted to evaluate the proliferating potential of tumor cells. Mitotic index in the SFN-treated tumor was significantly lower, 1.53 ± 0.57, compared to the non-treated control group, 2.15 ± 0.13 indicating a notable decrease in proliferating potential of tumor cells (Fig. 1C, F). At the same time, SFN did not decrease the Ki-67 index, however, the deviation was high in both groups (Fig. 1D, F). H&E staining after SFN treatment revealed partially reduced size of areas of necrosis, and also reduced intensity of inflammatory cell infiltration and change in their type: decrease in lymphocytes and histiocytes and increase in the proportion of neutrophil granulocytes. There were no features of anaplastic foci in the tumor sections after SFN treatment in contrast to control samples, showing less malignant type, in contrast to the non-treated group (see Supplementary Fig. S1, S2 and Table S1 online).

To further investigate the SFN effect on malignancy of the tumor the number of metastases was calculated in the lungs. The mean number of metastases was lower after SFN treatment (34 vs. 23 counts per mouse), however due to the dispersion of the results the difference was rated as statistically insignificant (Fig. 1E).

### Overall mice welfare

**Figure 2:**
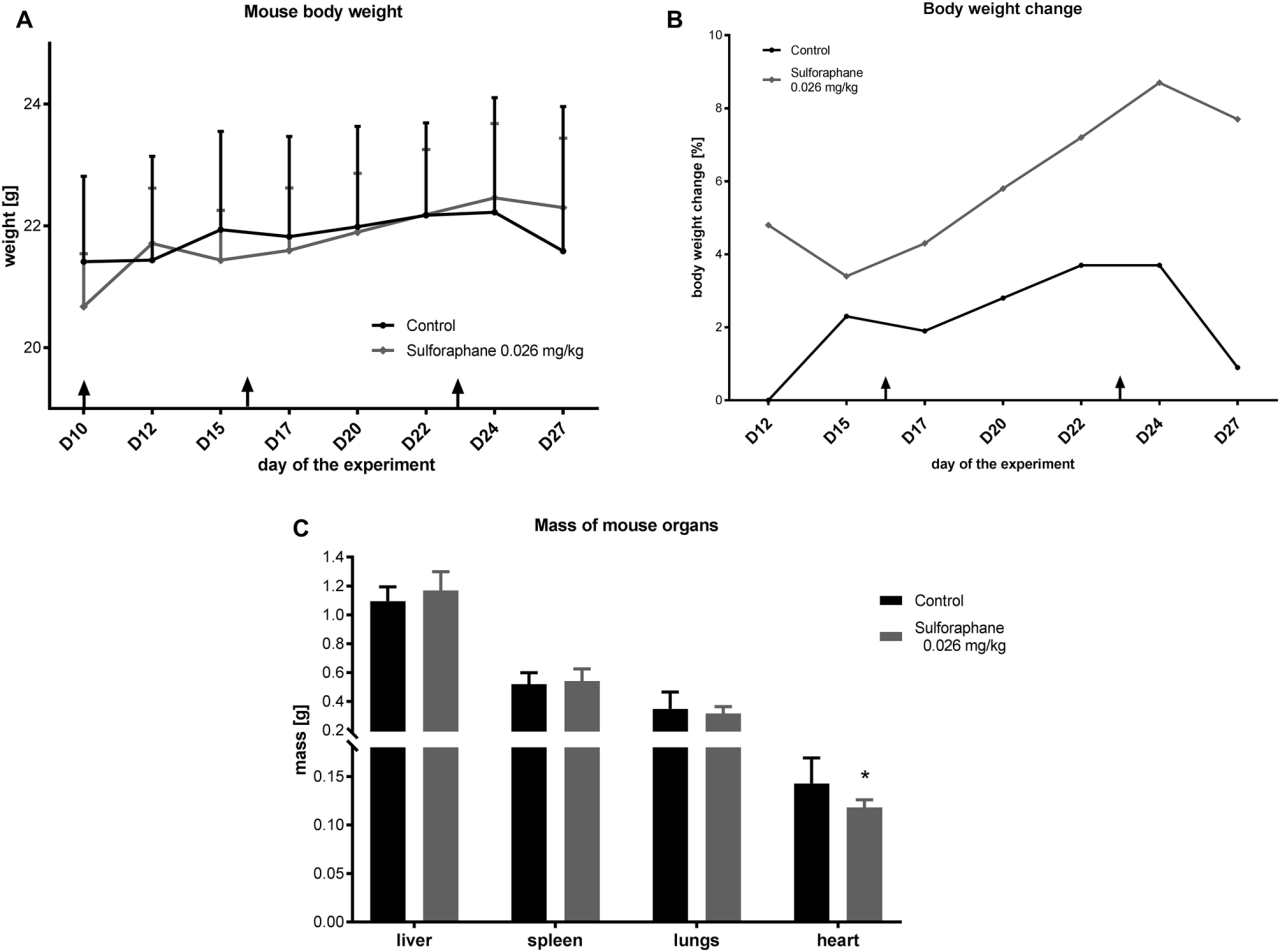
Toxicity of SFN in mice. (**A**) Body weight of mice, (**B**) mouse body weight changes curves during the time of the experiment. The arrows indicate the administration of SFN or injection water on days 10, 16, and 23. All data are presented from the control group and after administration of SFN (0.026 mg/kg) on days 10, 16, and 23 of the experiment as mean ± SD (n = 8). (**C**) A mass of mouse organs at the end of the experiment. Data is shown as mean ± SD (n = 8). *p < 0.05 vs control.

The welfare of mice was controlled during the time of the treatment. Most of the time of the experiment there was no weight loss observed. Only at the end of the experiment mice started to lose weight, but it was still higher than at the start (Fig. 2A, B).

The mass of the heart after the treatment was lower than in the control group (Fig. 2C). However, the biochemical analysis of blood parameters showed no significant change in the level of creatine kinase (CK) and isoenzyme MB (CK-MB) after SFN treatment (Table 1). A higher level of CK and its isoenzyme is an indicator of muscle tissue damage, mainly myocardial damage. There were no significant changes between the mass of the liver, spleen, and lungs (Fig. 2C).

**Table 1:** Blood morphology analysis of mice after 0.026 mg/kg SFN administration, control 4T1 bearing mice and healthy mice.

Group	Unit	Healthy mice (without tumor)	Control	SFN 0.026 mg/kg
Leukocytes	10^3^/μL	5.9 ± 1.5	319.8 ± 104.9	272 ± 147
Lymphocytes	10^3^/μL	4.73 ± 0.32	51.81 ± 12.47	53.04 ± 8.7
Monocytes	10^3^/μL	0.26 ± 0.06	31.66 ± 2.56	31.55 ± 2.99
Granulocytes	10^3^/μL	0.9 ± 0.35	236.33 ± 14.39	187.41 ± 11.42*
Lymphocytes	%	80.3 ± 5.4	16.2 ± 3.9	19.5 ± 3.2
Monocytes	%	4.4 ± 1	9.9 ± 0.8	11.6 ± 1.1*
Granulocytes	%	15.3 ± 6	73.9 ± 4.5	68.9 ± 4.2
Erythrocytes	10^6^/μL	8.1 ± 1.4	10 ± 1.7	10.6 ± 1.8
Hemoglobin	g/dL	13.8 ± 0.7	20.6 ± 3.2	21.6 ± 3.4
Hematocrit	%	39.2 ± 1.6	54.2 ± 9.2	56.9 ± 9.9
MCV	fL	48.3 ± 0.9	54.3 ± 2.1	53.9 ± 3
Platelets	10^3^/μL	482 ± 26.8	693 ± 239.8	753.1 ± 141.2
ALT	U/L	40.3 ± 4.1	24.2 ± 6.4	26 ± 9.7
AST	U/L	130.2 ± 25.3	169.2 ± 58.1	166.3 ± 76.2
Creatinine	μmol/L	8.3 ± 0.9	11 ± 4.3	7.3 ± 2.7
Urea	μmol/L	9.5 ± 2.3	7.9 ± 1.5	7.4 ± 1.2
Creatine kinase (CK)	U/L	No data	1326.9 ± 531.8	1326.5 ± 398.3
CK-MB	U/L	No data	411.1 ± 131.2	429.4 ± 77.9

The data represents mean values for each group ± SD (n = 8), * Mann–Whitney test vs control p < 0.05.

Blood morphology analysis revealed lower total leukocytes number and specifically a lower number of granulocytes, but higher platelets count compared to the control. There was also a significant increase in the percentage of monocytes compared to the control (Table 1).

### SFN cytotoxicity in vitro

**Figure 3:**
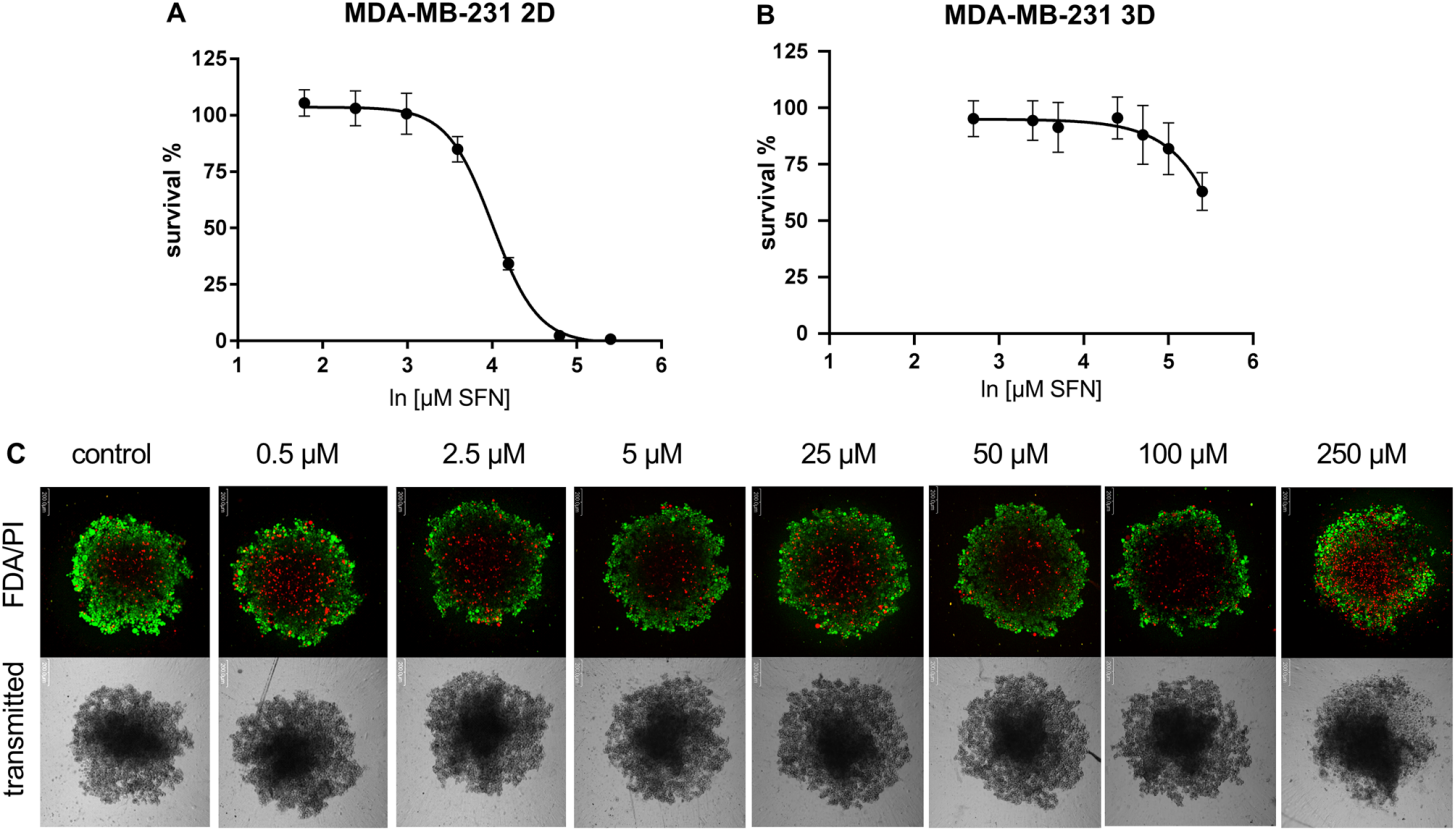
The cytotoxic activity of SFN in vitro. The survival curves of MDA-MB-231 cells from (**A**) 2D culture and (**B**) 3D spheroid culture after treatment with SFN (**A**) (0.06–250 µM) and (**B**) (0.5–250 µM), (**C**) microscopic images of the live/dead staining of MDA-MB-231 3D spheroid culture after SFN treatment (0.5–250 µM), where green—live cells (FDA), red—dead cells (PI). Scale bar 200 µm.

To evaluate the cytotoxicity of SFN, viability assays were performed on a 2D and 3D spheroid model of MDA-MB-231. The cytotoxicity on a 2D model showed that SFN acted hormetically—slightly boosted proliferation of the MDA-MB-231 cells at the lowest concentrations tested, but also effectively inhibited MDA-MB-231 cell growth at higher concentrations (5–250 µM) (Fig. 3A). Results gathered on Fig. 3B showed small to moderate SFN cytotoxicity on MDA-MB-231 spheroids. At a concentration of 250 µM, SFN lowered the survival to 65%, but at lower concentrations, it did not show a hormetic action—SFN at low concentrations (0.5–5 µM) did not boost the proliferation of cells forming a spheroid (Fig. 3B). The IC_50_ value on the 2D model for SFN was 10.56 ± 1.16 µM, while on 3D IC_50_ was higher than 250 µM.

The FDA/PI staining of spheroids showed differences in live/dead cells proportion after SFN treatment. At small concentrations of SFN, up to 2.5 µM, spheroids show small necrotic cores and high viability in the outer spheroid layer (Fig. 3C). At higher concentrations, starting from 5 µM, the viability of cells was significantly lower, and the number of dead cells and necrotic cores was growing. The morphology of spheroids has also changed. With higher concentrations, spheroids were more loosely aggregated and started to lose their circularity.

### In vitro cell migration/wound healing assay

**Figure 4:**
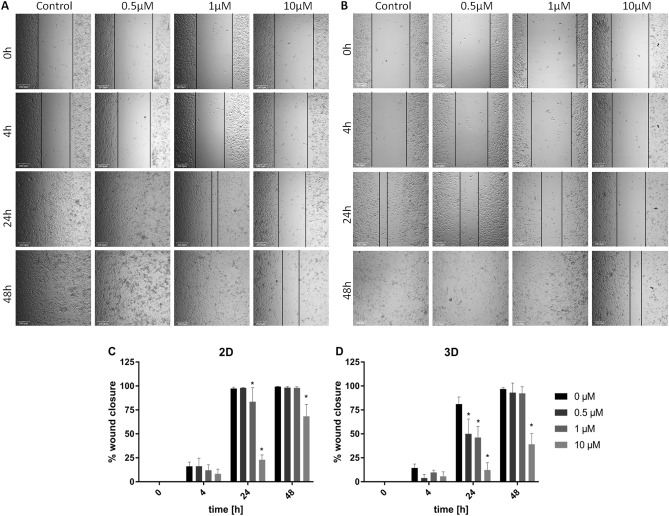
The wound healing assay after SFN treatment. Representative microscopic (×10) images of wound healing experiments from (**A**) 2D culture and (**B**) spheroid derived cells of MDA-MB-231 cell line and graphs representing the % wound closure from (**C**) 2D culture and (**D**) spheroid derived cells after 0.5 µM; 1 µM and 10 µM SFN treatment compared to control cells. Scale bar 200 µm. Data is shown as mean ± SD (n = 5). Asterisk: < 0.01 vs control.

To further assess the influence of SFN on cell migration, a wound-healing assay was performed. This assay was performed to explore the ability of this anticancer compound to impact the migration and formation of new cell–cell interactions, and the results show susceptibility to metastasis formation. The results gathered in Fig. 4A, C confirmed that in cells cultured in 2D monolayer culture only after 10 µM SFN treatment, the migration of cells was significantly inhibited, and in a smaller manner after 1 µM treatment. A wound healing assay was also performed on monolayer culture of cells derived from 3D spheroids cultures to investigate whether the 3D culture affects cell invasion and migration and better mimics the in vivo conditions (Fig. 4B, D). Spheroid-derived cells have not shown a higher migration rate compared to 2D cells albeit 1 and 10 µM SFN inhibited the wound healing in a greater manner than in 2D cultured cells (Fig. 4B, D). Furthermore, the inhibition of tumor cell migration was also observed after 24 h on cells treated with 0.5 and 1 µM SFN which was not observed in a wound healing assay performed on a 2D cultured cells. The results indicate that SFN can inhibit migration of cells only at higher concentrations (1 and 10 µM) tested however the spheroid-derived cells are more susceptible to SFN compared to the 2D cultured cells.

## Discussion

Breast cancer stands as a predominant cause of mortality among women on a global scale. Current therapeutic approaches remain inadequately effective. A promising approach to cancer prevention focuses on a healthy lifestyle that includes an anti-inflammatory diet and stress reduction. Accordingly, the growing popularity of dietary supplements is observed. Among these supplements, phytochemicals, like SFN, have garnered significant attention. These compounds are recognized for their dual attributes as chemopreventive agents, capable of impeding cancer onset, and as anticancer agents for treating established malignancies.

However, it is important to note that the quantities of phytochemicals administered through supplements exhibit substantial variation, and their bioavailability is often relatively limited. Consequently, the resultant concentrations of supplemented compounds in the bloodstream remain notably modest. In the case of SFN, its serum concentrations after broccoli consumption were reported to reach 0.94–2.27 μM^19^. Additionally, many phytochemicals, including a popular supplement—SFN, are considered hormetic compounds, meaning they exert divergent effects at low and high concentrations.

Most of the research is focused on high concentrations of SFN which acts as an anticancer compound. Very few reports focus on the antitumor effects of low concentrations of SFN, which are obtained mostly from sulfur-rich vegetables and supplement consumption. In this work, we focused on the 0.026 mg/kg dose of SFN administered intraperitoneally to mice with developed TNBC, which corresponds to a 1.5 μM SFN concentration in the mouse plasma. The results obtained in this study indicate that this concentration exhibited pharmacological, anticancer effects in the in vitro and animal in vivo models of TNBC. To our best knowledge this record provides the first evidence of TNBC growth and lung metastases inhibition in vivo by low, corresponding to dietary, doses of SFN.

There are a few reports in different TNBC models in vivo*,* however treated with 50 mg/kg SFN daily showing tumor growth inhibition. For example, Zhou et al.^20^ and Castro et al.^21^ obtained, respectively, over 50% and 14% reduction in tumor growth with daily 50 mg/kg, on a human TNBC model in mice . In contrast, the lower administered doses of SFN (2.5 and 5 mg/kg daily) promoted the carcinogenic effects of *N*-butyl-*N*-(4-hydroxybuty)l-nitrosamine on mice with bladder cancer^22^. The results obtained by us of the in vivo experiment showed up to 31% tumor growth inhibition (TGI) in the TNBC mouse model treated with a low, 0.026 mg/kg SFN weekly dose. Hence, the studied model assuming the lowest dose administered weekly showed up to be effective in tumor growth limitation providing new information on the dose–response profile of SFN.

The in vitro studies on a human TNBC 2D and 3D model were conducted to evaluate the mode of SFN action—whether SFN acts through the cytotoxic effect or the inhibition of metastasis (i.e., proliferation or migration). We chose to use not only 2D cell culture, which lack the complex three-dimensional architecture and cell–cell interactions found in vivo but also 3D in vitro cultures, such as spheroids that in contrast to 2D culture show a variety of malignant tumor characteristics in vivo, such as hypoxia, reduced proliferation, superior epithelial–mesenchymal transition (EMT), and elevated resistance to toxicological response^24^. SFN at low concentrations shows a stimulating effect on cell proliferation in 2D culture, but it did not stimulate 3D cell proliferation, which is relevant to the situation of developing tumors in the body. At higher concentrations, starting from 5 μM, SFN shows a cytotoxic effect on the 2D TNBC model (IC_50_ = 10.56 ± 1.16 µM), while on 3D SFN does not show cytotoxicity up to 250 µM indicating elevated resistance of 3D structures to SFN. At the same time, in in vivo model, already at low concentrations, SFN has shown an inhibitory effect on tumor growth, which is not observed in the in vitro model. When considering the effect in the in vivo model, one must take into account the described above malignant tumor characteristics, such as hypoxia, reduced proliferation, superior epithelial–mesenchymal transition (EMT) and also the entire tumor microenvironment (TME) that is a complicated system composed, apart of tumor cells, with infiltrating immune cells (such as macrophages and lymphocytes), the extracellular matrix and multiple signalling molecules. What is interesting, SFN has already been shown to inhibit EMT, and also to modulate the immune system^23^. Hence, the above results of SFN impact on cell proliferation and tumor size suggest that SFN action extends beyond direct impact on tumor cells.

A similar conclusion can be drawn from the analysis of SFN effect on metastasis and migration of TNBC cells. Antimigration and antimetastatic activity is an important aspect of the antitumor compound. SFN showed the potency to inhibit cell migration already at low concentrations, in vivo and in cells derived from spheroids, but not in 2D in vitro culture. In vivo results showed that SFN inhibited lung metastases formation compared to control, which is the major cause of breast cancer-related mortality. It can be associated with EMT inhibition by SFN due to cadherin and vimentin expression modulation and inhibition of EMT-related transcription factors^3^. Zhang et al. demonstrated that SFN inhibits the metastasis of TNBC cells by targeting the serine/threonine-protein kinase/mitogen-activated protein kinase extracellular signal-regulated kinase (RAF/MEK/ERK) signaling pathway to inhibit the formation of actin stress fibers and Jeong et al. demonstrated that by suppressing matrix metalloproteinase 9 activity, an effect achieved by blocking the focal adhesion kinase (FAK)/ERK/Akt kinase and Nuclear factor κB/ activator protein 1 (NF-κB/AP-1) pathways^25,26^.The in vivo study also confirms dual impact of SFN, precisely—on tumor cells and immune cells that comprise tumor microenvironment. Firs of all the histopathological analysis of tumors revealed that low SFN concentration exhibited antimitotic activity, as previously reported by us^27^.Apart from observed inhibition of the proliferating potential of tumor cells, the histological analysis of tumors also revealed a decreased intensity of inflammatory immune cell infiltration and a change in their type: a decrease in lymphocytes and an increase in the proportion of neutrophil granulocytes. At the same time in the peripheral blood, their population decreased compared to the control. In contrast to acknowledged beneficial impact of lymphocytes presence in tumor microenvironment, the role of neutrophils in cancer is ambiguous. In the tumor microenvironment, neutrophils can inhibit tumor progression by generating anti-tumor factors such as ROS, but more commonly, neutrophils have been reported as tumor accomplices in promoting cancer progression and metastasis by regulating tumor survival and migration, immune response, and angiogenesis^27,28^. Treatment with dietary dose of SFN induced neutrophil accumulation in tumor, suppressing tumor growth through direct cytostatic and/or cytotoxic effects and at the same time lowered the blood circulating granulocytes. SFN is a known activator of the Nuclear factor erythroid 2-related factor 2 (Nrf2), which plays a key role in regulating ROS secretion in neutrophils^29^. Therefore SFN can protect from extensive activation of neutrophils in peripheral blood, inhibiting neutrophil extracellular traps formation and tumor metastasis^30^. Treatment with a dietary dose of SFN can act by mobilizing neutrophils to the primary tumor, simultaneously reducing the blood-circulating granulocytes population through the Nrf2 pathway activation, thereby promoting the elimination of both cancer and metastatic cells. Therefore, at low concentrations in in vitro studies, this antitumor activity was not apparent because the models did not include immune cells.

In summary, these findings highlight the anti-tumor activity of low concentrations of SFN, demonstrating not only a direct anti-proliferative effect, but also pointing at a complex non-direct activity such as the modulation of tumor microenvironment and EMT, ultimately leading to tumor suppression and inhibition of metastasis.

## Conclusion

In conclusion, SFN at low concentrations led to the reduction of tumor volume together with decreased mitotic activity and reduction of metastases in animal TNBC model. This is consistent with the observed impaired motility of cells from SFN-treated spheroids. Moreover, the mobilization of neutrophils to the primary tumor, coupled with a reduction in the blood-circulating granulocytes collectively contributed to tumor eradication and a reduction in the extent of lung metastases. Considering that antiproliferating and antimetastatic activities were evident in the 3D in vitro and in vivo models, but not in the 2D in vitro model, the results strongly indicate that low SFN concentration acts not only through affecting directly cancer cell proliferation but also other cancer-related elements, i.e. components of the tumor microenvironment such as immune cells and EMT process.

### Supplementary Information

Supplementary Information.

## Abbreviations

ALT: Alanine aminotransferase
AST: Aspartate aminotransferase
CK: Creatinine kinase
CK-MB: Creatinine kinase isoenzyme MB
DOX: Doxorubicin
EMT: Epithelial–mesenchymal transition
H&E: Hematoxylin & eosin
IP: Intraperitoneally
PI: Propidine iodide
ROS: Reactive oxygen species
SFN: Sulforaphane
TGI: Tumor growth inhibition
TME: Tumor microenvironment
TV: Tumor volume
TNBC: Triple-negative breast cancer
