# Supplementary material for: Antitumor and antimetastatic effects of dietary sulforaphane in a triple-negative breast cancer models
Source: Sci Rep. 2024 Jul 11;14:16016. doi: 10.1038/s41598-024-65455-w (PMC11239657; doi:10.1038/s41598-024-65455-w)
Supplement: Supplementary file 1 — Supplementary Information. [file 41598_2024_65455_MOESM1_ESM.docx]

Supplementary Information to „Antitumor and antimetastatic effects of dietary sulforaphane in a triple-negative breast cancer models.”

A. Pogorzelska^a,1^*, M. Świtalska^b,2^, J. Wietrzyk^b,3^, M. Mazur^c^, M. Milczarek^a,4^, K. Medyńska^d^, K. Wiktorska^d,5^*

^a^Department of Biomedical Research, National Medicines Institute, Chełmska 30/34, Warsaw 00‑725, Poland,

^b^Laboratory of Experimental Anticancer Therapy, Hirszfeld Institute of Immunology and Experimental Therapy, Polish Academy of Sciences, Rudolfa Weigla 12, Wrocław 53‑114, Poland,

^c^Department of Chemistry, University of Warsaw, Ludwika Pasteura 1, Warsaw 02‑093, Poland,

^d^Department of Physics and Biophysics/Institute of Biology, Warsaw University of Life Sciences, Nowoursynowska 159, Warsaw 02‑776, Poland,

corresponding authors: a.pogorzelska@nil.gov.pl; katarzyna_wiktorska@sggw.edu.pl;

**Supplementary Table S1** A summary of histopathologic analysis of tumor sections.

| Control | SFN |
| --- | --- |
| Epithelial carcinomas of the mammary gland with a low degree of differentiation, showing minimal features of ductal structures. The foci of morphological features of anaplastic carcinomas with a rich tumor stroma. Extensive areas of necrosis/apoptosis (most likely due to intense tumor growth, resulting in hypoxia and malnutrition of the intensely dividing tumor cells). The stroma rich in spindle cells most likely of connective tissue, gaping blood vessels, hemorrhages and a rich mixed inflammatory cell infiltrate.  All the described features indicate a high histological/biological malignancy of the studied tumor and may suggest a high metastatic potential. | No significant differences in the histopathological features regarding the type of cancer. Partially reduced size of necrotic areas were visualised. Reduced intensity of inflammatory cell infiltration and a change in their type: a decrease in lymphocytes, and an increase in the proportion of neutrophil granulocytes. No features of anaplastic foci. |

Supplementary Table S2 Scoring of the histological appearance of tumors after sulforaphane (SFN) treatment and control group based on 4 tissue sections taking into account presence of anaplastic foci, cell polymorphism, presence of necrosis/apoptosis, lymphocytic infiltration and neutrophil infiltration.

|  | Control | | SFN | |
| --- | --- | --- | --- | --- |
|  | total | mean | total | mean |
| Anaplastic foci | 9 | 2.25 | 0 | 0 |
| Cell polymorphism | 7 | 1.75 | 11 | 2.75 |
| Necrosis/apoptosis | 15 | 3.75 | 15 | 3.75 |
| Lymphocytic infiltration | 13 | 3.25 | 7 | 1.75 |
| Neutrophil infiltration | 10 | 2.5 | 15 | 3.75 |


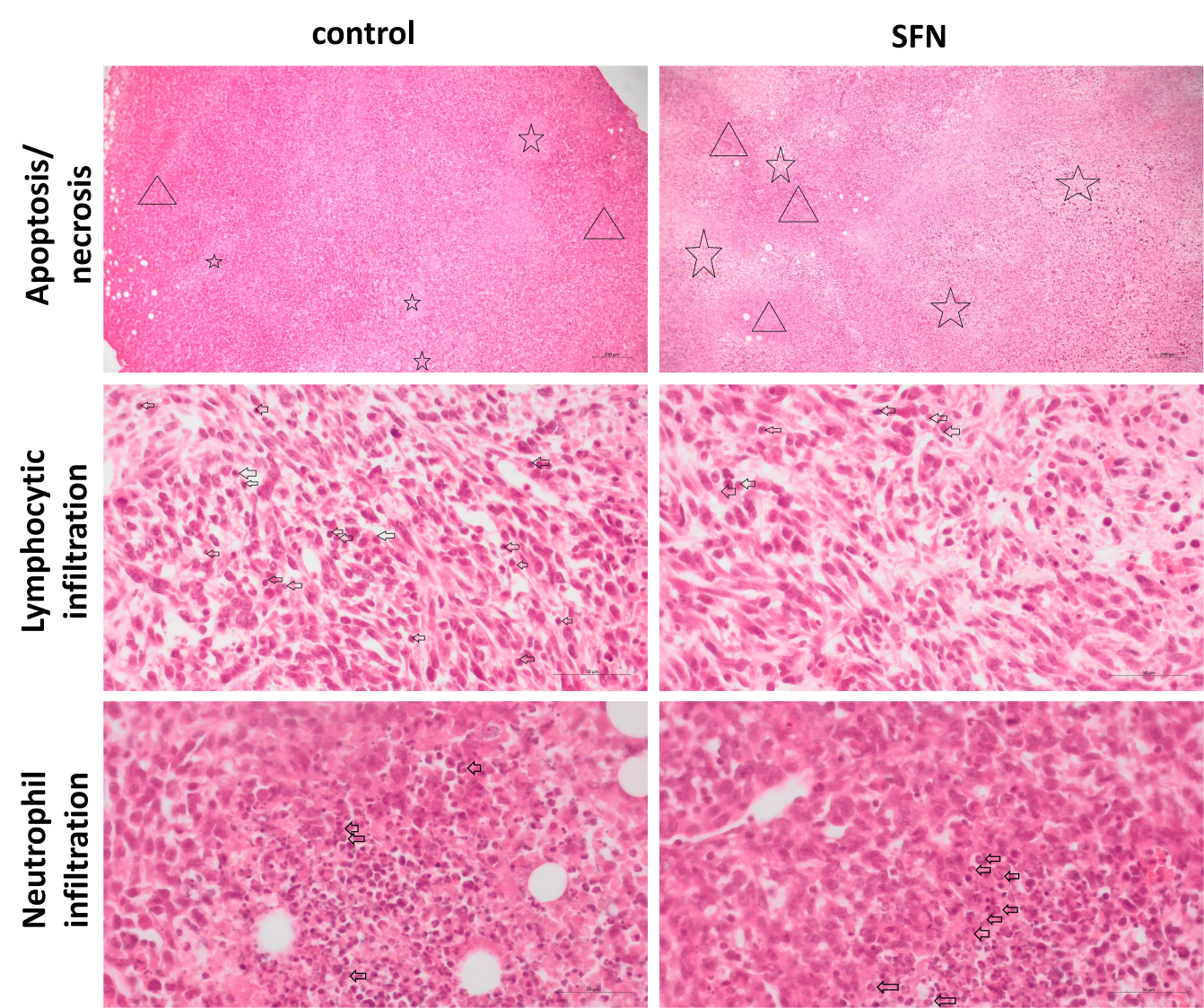


Supplementary Figure S1 Representative microscopic images of hematoxylin & eosin histochemical staining of tumor sections after Sulforaphane (SFN) treatment and control group, where star marks apoptosis/necrosis area; triangle – anaplastic foci and/or severe pleomorphism (scale bar 200µm), and arrows lymphocytic and neutrophil infiltration respectively (scale bar 50µm).
